# Why is adiabatic compressed air energy storage yet to become a viable energy storage option?

**DOI:** 10.1016/j.isci.2021.102440

**Published:** 2021-04-16

**Authors:** Edward R. Barbour, Daniel L. Pottie, Philip Eames

**Affiliations:** 1 Centre for Renewable Energy Systems Technology, Loughborough University, Loughborough, UK

**Keywords:** Energy Systems, Energy Management, Energy Storage

## Abstract

Recent theoretical studies have predicted that adiabatic compressed air energy storage (ACAES) can be an effective energy storage option in the future. However, major experimental projects and commercial ventures have so far failed to yield any viable prototypes. Here we explore the underlying reasons behind this failure. By developing an analytical idealized model of a typical ACAES design, we derive a design-dependent efficiency limit for a system with hypothetical, perfect components. This previously overlooked limit, equal to 93.6% under continuous cycling for a typical design, arises from irreversibility associated with the transient pressure in the system. Although the exact value is design dependent, the methodology we present for finding the limit is applicable for a wide range of designs. Turning to real systems, the limit alone does not fully explain the failure of practical ACAES research. However, reviewing the available evidence alongside our analytical model, we reason that underestimation of the system complexity, difficulty with the integration of off-the-shelf components, and a number of misleading performance claims are the primary reasons hindering ACAES development.

## Graphical abstract

**Figure:**
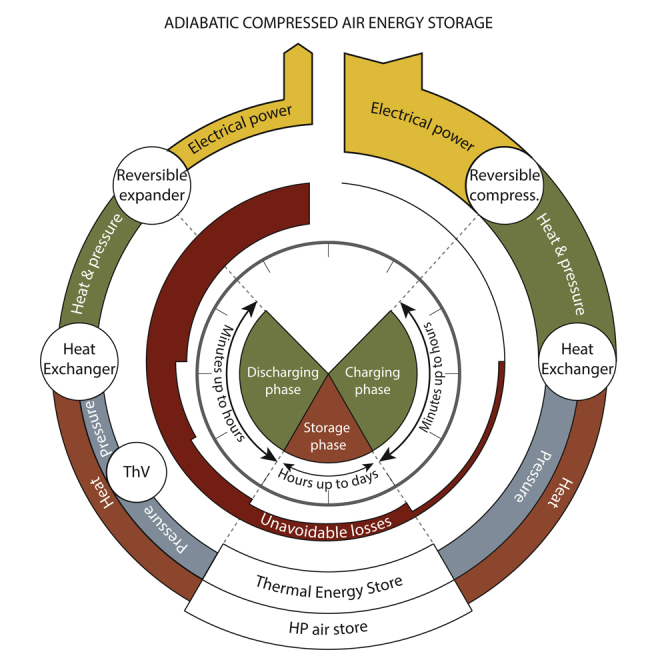

## Introduction

**Figure 1:**
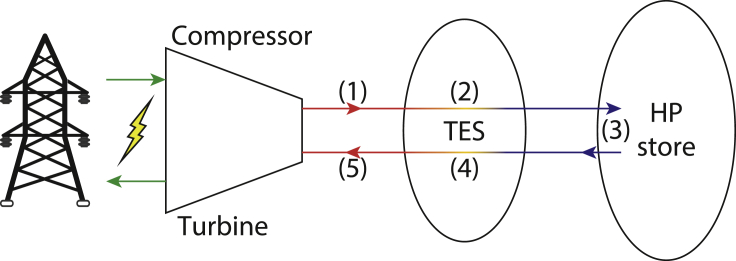
ACAES system definition Thermo-mechanical processes that define ACAES.

Adiabatic compressed air energy storage (ACAES) is a concept for thermo-mechanical energy storage with the potential to offer low-cost, large-scale, and fossil-fuel-free operation. The operation is described simplistically as follows. To charge the system, work is used to compress atmospheric air in compressors (Figure 1 point (1)), generating heat in the process. The heat at the compressor outlets is removed from the air via heat exchangers (HEX) and stored in separate thermal energy stores (TES) (Figure 1 point (2)), whereas the cool compressed air is stored in a high-pressure (HP) air store (Figure 1 point (3)). To discharge the system, the cool compressed air is recombined with the heat from the TES to generate hot, high-pressure air (Figure 1 point (4)), which is expanded through turbines to generate work (Figure 1 point (5)). Figure 1 depicts the process.

Despite having a very similar name, ACAES is distinct from current compressed air energy storage (CAES) plants, which are diabatic. Two utility-scale CAES plants—Huntorf, DE (321MW) and MacIntosh, USA (110MW)—have existed since 1978 and 1991 respectively, using salt caverns as underground storage (Crotogino et al., 2001; Hounslow et al., 1998). These systems also charge by using work to compress atmospheric air, generating heat in the process; however, the heat is wasted and exergy is stored only in the cool pressurized air. When the system is discharged, this air is heated using fossil fuel (typically natural gas) and is used to drive a turbine. Hence, this system arguably has more similarity with gas turbine technology than a pure energy storage plant. Rather, the major difference between CAES and a gas turbine is the temporal decoupling of the compressor and turbine operation, which requires the storage of compressed air. As such, CAES has significant associated emissions (these are estimated at $228g_{CO_{2}}/kWh$ when charged with wind energy—60% of the emissions reported for gas turbines [Mason and Archer, 2012]) and cannot be considered solely as an electricity storage system, such as batteries or pumped hydroelectric storage.

As a result of the shortcomings of CAES and due to the appeal of a purely thermo-mechanical energy storage system, with no reliance on either fossil fuel or rare materials, much recent research has focused on ACAES. Most of these studies use numerical thermodynamic models (Grazzini and Milazzo, 2008; Barbour et al., 2015; Sciacovelli et al., 2017) or (generally small-scale) experimental work augmented with numerical aspects to predict the electrical-to-electrical efficiency, typically in the range 50%–75% (Grazzini and Milazzo, 2008; Barbour et al., 2015; Sciacovelli et al., 2017; Szablowski et al., 2017; Peng et al., 2016). The exact value predicted depends on the assumed performance of the constituent components as well as the precise system configuration, with lower estimates typically taking a more pessimistic view of component performance. However, the thermodynamic performance limits of ACAES are under-explored and it should be noted that no prototype system has attained the predicted performance.

**Figure 2:**
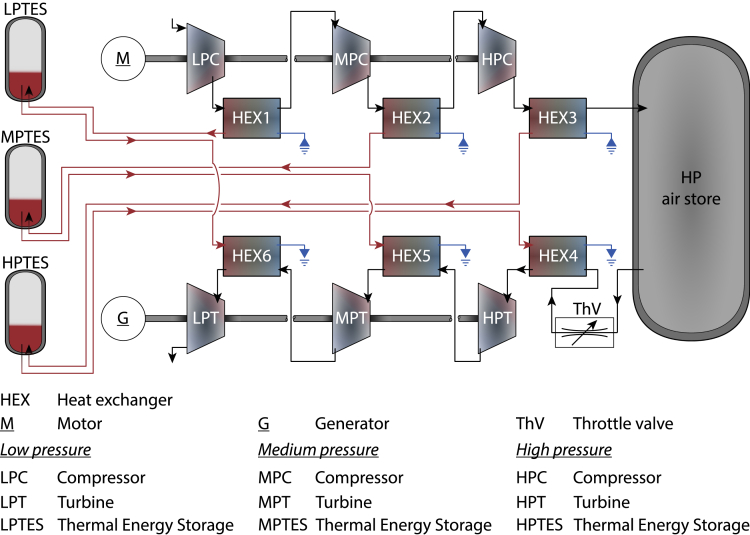
Case study ACAES system A schematic of an ACAES system that belies the complexity. Purely adiabatic compressors with variable compressor ratios must operate efficiently. Cooling stages should have minimal pressure drop and maximum effectiveness. Heat and mixing losses in the TES and HP store should be minimized. Steady flow should be maintained during expansion, and heating stages should exactly reverse the cooling stages. Effective integration between components is crucial and control must be maintained under all conditions. For this system and assuming hypothetical perfectly ideal components we find that 6.4% of the input work is unrecoverable.

Here, we demonstrate here that the efficiency limit of typical designs is considerably lower than 100%—indeed, for the design illustrated in Figure 2 we find a limit of 93.6%. Although the exact limit is specific to the design proposed, our methodology can be applied to designs with differing numbers of stages and the loss mechanisms are the same. The limit arises due to exergy destruction as heat at different temperatures is mixed in the system and the exhaust, as well as throttling losses. This unavoidable penalty has been overlooked by previous research, and although by itself it does not explain the failure of ACAES development to yield a viable design, it highlights the target component operation for ACAES to reach maximum performance. Comparing this operation to that available from conventional components and analyzing the available literature in the scientific and public domains, we postulate that three additional issues have hindered ACAES development. These are as follows. (1) Underestimation of the real process complexity and the misconception that the system can be built with *off-the-shelf* compressors, turbines, and heat exchangers. (2) Misleading efficiency claims from early-stage commercial projects, often repeated in academic review articles, and unrealistic assumptions in previous modeling work, which are rarely challenged. (3) A combination of the reasons (1) and (2) leading to over-ambitious development in commercial ventures, where any lessons learned are hidden, in turn stifling much-needed transparent experimental projects. Furthermore, the high investment costs of prototype ACAES systems are a major challenge, although this is a common hurdle across thermo-mechanical energy storage development.

We therefore recommend the following three actions to improve the prospects of ACAES as a potential large-scale energy storage option. (1) Further transparent experimental work on ACAES in universities, which is crucial for understanding detailed system performance. (2) Where commercial projects are funded with majority public money, a minimum threshold of documentation should be stipulated, which, in the event of project termination, must include the reasons for failure to achieve the stated aims. (3) High-quality research and funding should not be influenced by hearsay or unverifiable performance claims; in particular, opaque performance claims with a lack of documentation for commercial sensitivity reasons should not influence academic research until verified.

## Results

### Thermodynamic limits

We now discuss the efficiency limits of the typical ACAES design as shown in Figure 2. The purpose is to reveal both the theoretical efficiency limit and the corresponding operation of the system components. Accordingly, we consider the limit in which all components are ideal and reversible (except for the throttle valve, which is ideal but not reversible) and we treat air as a dry, ideal gas with constant specific heats $c_{p}$ and $c_{v}$. The HP air store has a constant volume (i.e., isochoric), and during the charging period, air is added at ambient temperature because perfect counter-current exchangers are assumed. We treat the HP air store as adiabatic during the charge and discharge processes, so the air compression during charging leads to a significant temperature increase. This is justified by the need for high tensile stress tolerance in the HP store for operation and safety, meaning a store with a large surface-area-to-volume ratio will not be practical. We also note that real CAES systems experience significant temperature rise during charging and temperature drop during discharging (Crotogino et al., 2001; Raju and Khaitan, 2012) and similar temperature behaviors are experienced in experiments on ACAES systems (Geissbühler et al., 2018; Wang et al., 2016a). During the idle periods (between charging/discharging and discharging/charging) we consider two limiting cases, an adiabatic store (denoted AD) in which no heat is exchanged with the environment and a store that undergoes temperature recovery (denoted TR) to the ambient temperature. Under these assumptions, the charging work is given by Equation 1 (see methods).

(Equation 1) $$W^{chg}=N\frac{V^{st}c_{p}p_{i}^{chg}}{R\gamma }\left\{\frac{1}{\frac{\gamma -1}{N\gamma }+1}\left[\frac{p_{f}^{chg}}{p_{i}^{chg}}{\left(\frac{p_{f}^{chg}}{p^{0}}\right)}^{\frac{\gamma -1}{N\gamma }}-{\left(\frac{p_{i}^{chg}}{p^{0}}\right)}^{\frac{\gamma -1}{N\gamma }}\right]+1-\frac{p_{f}^{chg}}{p_{i}^{chg}}\right\}$$

In Equation 1, pressures $p_{i}^{chg}$ and $p_{f}^{chg}$ are the initial and final HP store pressures, respectively, during the charge and *N* is the number of compression stages (each stage has equal compression ratio). The parameter γ is the ratio of the specific heats at standard conditions and signifies compression along an isentropic reversible path. Equation 2 gives the temperature in the HP air store as a result of the air compression within (see methods).

(Equation 2) $$T_{f}^{chg}=\frac{\gamma T^{0}}{\frac{p_{i}^{chg}}{p_{f}^{chg}}\left(\gamma \frac{T^{0}}{T_{i}^{chg}}-1\right)+1}$$

$T_{i}^{chg}$ is the initial store temperature for the charging process, which is equal to the ambient for the first cycle. The compression work is stored as exergy partly in the HP air store and partly in the TES (Budt et al., 2016). Although there is a separate TES for each compression stage—because imperfect heat exchange leads to successive increases in compressor outlet temperature—with perfect heat exchange, the temperature of each TES is the same. The compressor outlet temperatures are variable and are a function of the instantaneous pressure ratio and the inlet air temperature. In the limit of perfect heat exchange and isentropic compression, the TES temperature is the average compressor outlet temperature, as expressed by Equation 3 (see methods).

(Equation 3) $$T^{TES}=\frac{T^{0}p_{i}^{chg}}{\left(\frac{\gamma -1}{N\gamma }+1\right)\left(p_{f}^{chg}-p_{i}^{chg}\right)}\left[\frac{p_{f}^{chg}}{p_{i}^{chg}}{\left(\frac{p_{f}^{chg}}{p^{0}}\right)}^{\frac{\gamma -1}{N\gamma }}-{\left(\frac{p_{i}^{chg}}{p^{0}}\right)}^{\frac{\gamma -1}{N\gamma }}\right]$$

This also provides the maximum reheat temperature available during discharge, assuming no TES cooling during the idle period. Hence the maximum recoverable work is:

(Equation 4) $$W^{dis}=N\frac{c_{p}T^{TES}V^{st}}{RT_{i}^{dis}}\left(1-{\left(\frac{p^{thr}}{p^{0}}\right)}^{\frac{1-\gamma }{N\gamma }}\right)\left[p_{i}^{dis}-p^{thr}{\left(\frac{p^{thr}}{p_{i}^{dis}}\right)}^{\frac{1-\gamma }{\gamma }}\right]$$

In Equation 4, $p_{i}^{dis}$ and $T_{i}^{dis}$ are the initial discharge pressure and temperature of the store while the minimum pressure is the throttle pressure $p^{thr}$. The recovered work clearly depends on the throttle pressure, which regulates the pressure entering the turbines, allowing design-point operation. This increases their reliability and efficiency (Sciacovelli et al., 2017; Zhang et al., 2019; He et al., 2017) at the necessary cost of some exergy destruction due to the air entropy change through the throttle. The round trip efficiency of the system is given by:

(Equation 5) $$\eta^{RT}=\frac{W^{dis}}{W^{chg}}$$

**Figure 3:**
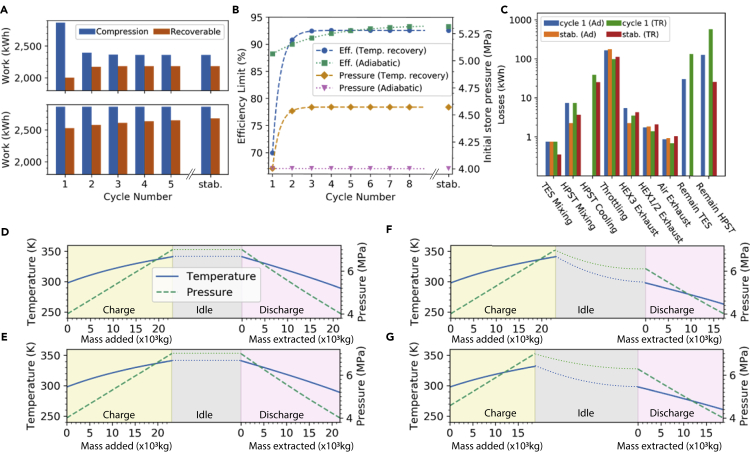
Summary of performance and results Efficiency limits for the proposed ACAES system (1,000 m^3^). (A) Compression and recoverable work over five cycles and for a stabilized cycle (stab.). (B) Efficiency limit and initial store pressure over 10 cycles. (C) Exergy accounting for the adiabatic store (denoted AD) and temperature recovery (denoted TR) systems. (D–G) Illustration of the pressure and temperature changes during the charging, idle, and discharge periods for (D) the first cycle with AD store, (E) a stabilized cycle with AD store, (F) the first cycle TR store, and (G) a stabilized cycle with TR store. The differences between (D) and (E) and (F) and (G) illustrate how the mass of air added/extracted from the HP store equalizes compared with the initial cycle. Temperature and pressure in the idle period are qualitatively illustrated.

The implications of Equations 1, 2, 3, 4, and 5 are illustrated in Figure 3A. We take the base case for our typical system as a $1,000m^{3}$ store with minimum pressure 4MPa, maximum pressure 7MPa, N=3 compression and expansion stages, and air throttled to 4MPa before expansion. These pressures limits are similar to the conditions at Huntorf and McIntosh and those assumed in previous studies (Sciacovelli et al., 2017; Zhang et al., 2019; He et al., 2017). With these assumptions we find an efficiency limit of 88.2% for the first cycle, which rises to 93.6% (as calculated by Equation 5) under continuous cycling for the adiabatic (AD) store (see Figure 3B). With continuous cycling we assume that the mass of air in the HP store remains constant between the end of the discharge of the previous cycle and start of the charge in the next. Hence in the AD case the pressure and temperature remain unchanged during the time between cycles, whereas in the TR case the pressure at the start of the next cycle is higher as the store temperature recovers to the ambient. We note that it takes approximately five cycles for the mass to stabilize for the TR case and 20 cycles for the AD case.

The results are summarized in Table 1. The exergy accounting for both systems is shown in Figure 3C, with the full analytic exergy accounting method described in supplemental information section S1 and summarized in Table S1 (the method is general for any number of stages). Although the efficiency limit of both systems (AD and TR) under continuous cycling is high, it must be emphasized that these are idealized limits. That is explicitly to say that they represent the *design maximum possible efficiency*, so 6.4% of the input exergy is unrecoverable even with perfectly ideal components in the AD case, rising to 7.5% for the TR case.

**Table 1:** Calculated efficiency limits for isochoric idealized ACAES

	HP store heat regime	
	Adiabatic (AD)	Temperature recovery (TR)
First cycle	88.2%	69.9%
Continuous cycling	93.6%	92.5%

For the AD case, direct throttling losses are the largest loss, and the first cycle is also restricted by the large proportion of air that cannot be extracted from the store due to the discharge temperature drop (Figure 3D), leaving exergy remaining in the HP store and TES (Figure 3C). These effects combine to limit the first cycle efficiency to 88.2%. Once the mass has stabilized (Figures 3D and 3E), the efficiency rises to 93.6% and the throttling losses are dominant, with small mixing losses in the HP store, the TES, and exhaust streams. For the TR case, the first cycle efficiency is 69.9% due to the air mass that cannot be extracted due to the temperature drop. However, after five cycles the air mass added and extracted has stabilized and the initial storage pressure has increased to 4.57MPa, as can be seen in the difference between final temperature, pressure and mass added and extracted in Figures 3F and 3G, yielding an efficiency limit of 92.5%. Figure 3C shows that once stabilized throttling accounts for the majority of the exergy destruction in both the AD and TR systems. The throttling also increases downstream losses in the exhaust fluid streams; however, these are small in magnitude.

The exact efficiency limit revealed is design-dependent (although it does not depend on the store volume), as is illustrated by the differences between the AD and TR cases. However, it is widely applicable given the similarity between the majority of designs proposed in available literature and Figure 2. Furthermore, our approach is general for any number of stages. Importantly, the analysis reveals the complexity of idealized ACAES, demonstrating that even with highly efficient components the losses associated with thermal mixing will quickly become non-negligible. In real systems, the store will not be completely adiabatic during charging/discharging and thermal conduction through the walls will influence the air temperature. This effect will become more relevant as the power-to-energy ratio of the system decreases, and previous work has shown that caverns with large discharge times can deviate significantly from the adiabatic assumption (Raju and Khaitan, 2012). However, these studies also showed that this difference is small for relatively short charge/discharge times, which is likely applicable for artificial air stores with low surface-area-to-volume ratio.

### Challenges for ACAES components

There is a common misconception that the majority of components in the ACAES system shown in Figure 2 can be acquired “*off-the-shelf.*” Indeed, engineers are very familiar with industrial compressors, turbines, and HEX, and for many applications these items can be ordered as standard. However, for use in ACAES there are many crucial differences between the constituent components and their counterparts developed for previous applications. We discuss challenges for ACAES compressors, HEX, and expanders and highlight how they differ from readily available components. The HP store also has design challenges; however, in its simplest form a pressure vessel with suitable tolerance could be used.

### Compressors

In ACAES, the compressions should be adiabatic (i.e., isentropic and reversible), with all heat exchange taking place in the dedicated HEX that supplies the TES, otherwise the compression process will not follow a reversible path. In contrast, compressors for many other applications are designed to minimize the work input per unit production of high-pressure air, which typically involves simultaneous compression and cooling. This cooling of the compressor, while reducing the compression work, leads to additional irreversibility generation on top of the unavoidable frictional, leakage, and aerodynamic losses. The compressors in ACAES should also have high single-stage pressure ratios (>3) with high isentropic efficiency to achieve good energy density. These constraints make the use of axial compressors difficult because they are unable to reach higher pressure ratios without introducing sonic flow choke or having to design adequate convergent-divergent blade sections to achieve an efficient transonic transition. Moreover, the compressor operating principle causes the air flow to decelerate, creating an adverse pressure gradient, which can lead to stall and surge. This results in smaller allowable pressure ratios in compressors than axial turbines. These challenges are not only cost related, as more compression stages are required for the same pressure ratio, but also there are thermodynamic downsides arising from asymmetrical pressure distributions in charge/discharge. In contrast with axial machines, reciprocating compressors are able to reach the required pressure ratio without facing these issues, as the compression takes place on mostly stationary air. However, the mass flow capability of positive displacement machines is (relatively) smaller than axial, and ACAES charge requirements might lead to unpractical designs such as large piston diameters or several simultaneous chambers in parallel.

A further design challenge is that with isochoric air storage the compressors must function at high efficiency over a range of pressure ratios (Sciacovelli et al., 2017). This challenge is illustrated by recent studies with compressor models calibrated from experimental data, the results of which indicate that compressor performance deteriorates rapidly as the operation range increases (Chen et al., 2020; Ma et al., 2019). The range of pressure ratios encountered also necessitates that compressors operate with variable mass flow if constant power is to be achieved, which is further detrimental for performance (Sun et al., 2020). It is notable that poor compressor performance due to the range of pressure ratios encountered and unsteady mass flow rates is suggested as a primary reason for the low performance of prototype experimental ACAES systems (Wang et al., 2016a).

### Heat exhangers

The HEX required for ACAES are also highly non-standard and, like the compressors, must operate with variable mass flow, which is a design challenge (Zhang et al., 2019). Here, HEX1−3 are compressor aftercoolers while HEX4−6 are turbine preheaters (see Figure 2). The compressor coolant should enter the TES as close as possible to the compressor outlet temperature, necessitating a HEX temperature cross, which will be challenging to design cost effectively. In typical compressor aftercooler designs, heat transfer is enhanced by increasing the available heat transfer area and the air-to-coolant temperature difference. The effect of the former is limited by manufacturing techniques, cost restriction, and footprint available. To achieve the latter, however, the coolant mass flow rate is set considerably higher than the required mass flow for a balanced HEX, reducing the coolant temperature rise to maintain the thermal gradient with the air. This greatly improves the heat exchanger effectiveness; however, it reduces the process reversibility. The requirement for both cooling (during charge) and later reheating (during discharge) demands high process reversibility. This introduces a natural trade-off because (1) compressors operate better when the aftercooler coolant flow rate increases well above the balanced requirements but (2) to guarantee proper turbine inlet temperature, the coolant flow rate must meet the balanced requirements for the HEX. Therefore, ACAES heat exchangers need considerably larger contact areas than conventional aftercoolers, and it is of paramount importance to ensure that this does not lead to exceedingly large pressure losses in all HEX stages. The need to balance the HEX at all times is also a control challenge (Shah and Sekulic, 2003).

In general for ACAES, the number of cooling and heating stages is a design choice, with more stages leading to lower compressor outlet and TES temperatures. However, there are practical limits on the number of compressors and HEX it is possible to link in series without introducing severe pressure losses. Therefore most designs opt for between two and four stages, which predicates high temperatures at the compressor outlets, rendering even pressurized liquid water an impractical coolant. Hence various thermal oils have been suggested as coolant options (Pickard et al., 2009); however, these are expensive. Packed bed HEX offer potential for high-temperature heat storage with high effectiveness (Barbour et al., 2015; Peng et al., 2016) and would also mitigate some of the losses associated with thermal mixing in the TES; however, large tank sizes with high pressure tolerance may not be cost effective.

### Expanders

During discharge, the air expansion process is relatively similar to the operation of closed cycle gas turbines, because the working fluid (air) is externally heated using stored heat from the TES. However, the overall pressure ratio for the expansion is significantly higher than in typical gas turbines for power generation (typical gas turbines have an overall pressure ratio ∼10–30) (Sanjay et al., 2007; Horlock, 1995). Therefore, in the conventional CAES systems at Huntorf and MacIntosh, modified steam turbine technology is employed for the high-pressure expansion (Hounslow et al., 1998) and similar customized designs will be required in ACAES. Small-scale experiments have also adapted automotive turbocharge units (Maia et al., 2016); however, poor efficiency (peak instantaneous expansion-only efficiency of 45%) was reported. Although the need for custom design due to the high expansion inlet pressure will increase costs, the temperatures are lower than those encountered in gas turbines—maximum temperatures in modern high-efficiency gas turbines often exceed 1,800K (Sanjay et al., 2007) compared with >600K for the design shown in Figure 2. This is favorable from a costs perspective because designing materials to withstand these high temperatures is a significant source of expense in high-efficiency gas turbines and the lower temperatures may increase the power generation ramping rate.

Generally, designing turbines for ACAES is less of a challenge than compressors and HEX. A throttle valve installed upstream of the discharge turbo-machinery can control for a constant pressure ratio, so achieving efficient off-design operation is less critical. Moreover, the overall pressure gradient follows the main flow direction, thus allowing for greater pressure ratios over each expansion stage (compared with the compressors). This way, it is possible to have fewer expansion than compression stages, resulting in cost reduction. However, from a thermodynamic reversibility standpoint it is advantageous to match the compression and expansion paths.

### Storage

The simplest and most commonly suggested method of HP air storage consists of an isochoric reservoir, cycling between two set pressure levels as the system charges and discharges. Usually, this role is fulfilled by an underground cavern or artificial steel pressure vessel. Owing to the high pressure involved, the main design challenge is maintaining the necessary structural integrity over long lifetimes at low cost, which favors underground caverns for large-scale systems or smaller artificial stores with low surface-area-to-volume ratios. For underground air storage, the available geology must withstand not only the pressure requirements but also the impact of cyclical temperature fluctuations, potential liquid condensation (a particular challenge in salt-based geology), or any other chemical interactions. The Iowa Stored Energy Park (proposed 270-MW plant with more than $8 million investment) is an example of a project failure due to unsuitable geological conditions. Although appearing suitable at first, low sandstone permeability meant the geology was unable to sustain the required air mass flow (Schulte et al., 2012). Isochoric storage also influences the other components in the system, because the variations in pressure (and mass) in a constant volume mean that the other components must function with variable pressures. This, as discussed already, leads to several design challenges and the air compression within the store also results in thermal mixing (see Figure 3C), although this is generally a small loss.

The alternative to isochoric storage is isobaric storage. By altering the storage volume during the operation, the pressure (and temperature) variation can be mitigated, allowing components to operate at design-point. However, achieving isobaric operation is a major engineering challenge and only a few methods are proposed in the literature. These include sliding, movable barriers (Chen et al., 2018), liquid displacement (Mazloum et al., 2017) from/to an underground cavern to a body of water (e.g*.*, lake), and flexible underwater storage bags (Pimm et al., 2014). Overall, the question for isobaric storage is whether the increase in the performance of other components compensates for the increased complexity in the HP store design.

### Performance claims and current state of the technology

Despite two decades of research and significant funding, ACAES remains a technology squarely in early-stage research. To understand why, we review some major experimental studies and the available literature on pilot projects and commercial demonstration plants. These are summarized in Table 2.

**Table 2:** Major ACAES projects and experimental academic studies

Project	Status	Performance	Documented?	Notes
ALACAES	Underground tunnel air store and TES testing	Simulated 72%	Geissbühler et al. (2018)	The plant does not include a turbine so performance is simulated
ADELE	Project finished, no plant built	Claimed >70%	Zunft et al. (2017)	Planned construction never started due to unfavorable economic conditions
TICC 500	Completed pilot plant	Measured 22.6%	Wang et al. (2016a)	Five-stage reciprocating compressor. Low efficiency ascribed to unsteady operations
Lightsail	Commercial, insolvent	Claimed 90% *thermal efficiency*	No	Raised in excess of $70 million, water injection compression
SustainX	Commercial, insolvent	–	No	Liquid piston compression, US DOE invested $∼5.5 million. Pilot plant results never presented
Hydrostor	Commercial, two demonstration plants	–	Partially in Ebrahimi et al. (2019)	Isobaric water displacement storage, first pilot plant uses supplementary electric heater
1.5-MW pilot	Commercial?	Claimed 55%	No	Mentioned in Wang et al. (2017), functional references not provided
10-MW pilot	Commercial?	Claimed >60%	No	Mentioned in Wang et al. (2017), functional references not provided

We find that most academic studies undertake experiments on individual system components and subsequently use simulation to infer the whole-system performance, rather than experimental analysis on the complete system. For example, Geissbuhler et al*.* employ a large underground tunnel as an HP air store, testing the pressure integrity of the tunnel and the performance of the TES (Geissbühler et al., 2018). However, their efficiency estimate of 63%–74% is based on simple thermodynamic models of compressors and turbines with constant efficiency across the range of pressures encountered, which is unlikely to be realistic in a real system (Sciacovelli et al., 2017; Wang et al., 2016a). In another notable part-experimental study, sophisticated models of compressors and scroll expanders in small-scale ACAES were developed to fit with measured performance data. An efficiency of 13%–25% for a single-stage ACAES system was estimated, and it was suggested that this could improve to 60% for a 3-stage design (Chen et al., 2020). However, this efficiency relied on a pressure variation less than 0.05MPa, with the efficiency dropping rapidly as the pressure variation increased. It must also be noted that the system included no heat exchange—rather the expanding air was reheated to the maximum compression temperature in lieu of heat exchangers and a TES.

Overall, there is precious little published experimental work where work input and output from the system has actually been measured over a full cycle rather than inferred. The European Union-funded project ADELE aimed to build an ACAES plant with 70% efficiency (Zunft et al., 2017); however, no plant was ever built. Despite this, published literature from the project claimed success in confirming 70% efficiency as attainable with existing components (Zunft et al., 2017). The most notable experimental study on a complete ACAES system in the academic literature details a 500-kW ACAES pilot plant, documented in Wang et al. (2016a) and Mei et al. (2015). This plant achieved an electric-to-electric efficiency of 22% (Wang et al., 2016a), and a major reason for the poor performance was the unsteady operations of compressors and turbines caused by the HP store pressure variation (Wang et al., 2016a). Other studies that have measured work output in small-scale systems have struggled to achieve any reasonable efficiencies. For example, Cheayb et al*.* built a novel trigenerative CAES system and predicted that favorable efficiencies should be possible; however the measured electrical-to-electrical efficiency was only 3.6% (Cheayb et al., 2019). More often than not, experimental academic studies do not report the cycle efficiency even where this would be possible, instead focusing on metrics like peak instantaneous efficiency, which is defined within a very narrow pressure range.

**Figure 4:**
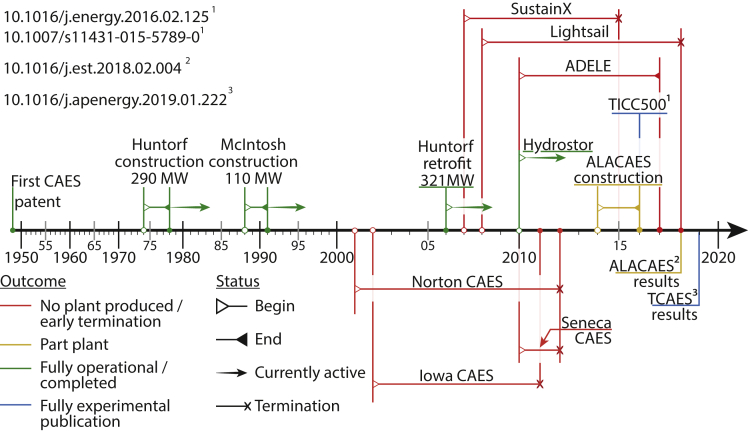
Timeline of major technology milestones Timeline of CAES/ACAES projects. Blue corresponds to recent prototypes in which energy consumption and generation are directly measured. Yellow are related to the ALACAES publications. Green marks completed or ongoing projects, including Huntorf and MacIntosh. Red shows major failed projects with early termination, bankruptcy, and no physical assemblies.

Outside of the academic studies, there has been significant hype around novel ACAES systems in early-stage commercial ventures, such as Lightsail Energy and SustainX; however, despite promising early press releases—Lightsail made claims of a thermal efficiency around 90% (Chen et al., 2016)—the majority of these companies have ceased trading. Lightsail Energy is a high-profile example of an ACAES startup that raised in excess of $70 million dollars Wesoff (2016) (accessed August, 2020); however, the company has now ceased operations. Despite this, Lightsail is still routinely cited in academic articles without mention that it no longer operates and failed to deploy a prototype. The Canadian venture Hydrostor is an exception that (in 2020) continues to operate with two demonstration ACAES projects and recently won a 2019 Energy Storage North America innovation award. Although technical details about Hydrostor projects are unavailable in the public domain, it is notable that Hydrostor runs an isobaric system using water displacement. This further evidences our suggestion that variable pressure operation is a major challenge for ACAES. Figure 4 provides a timeline of notable demonstration ACAES projects and major experimental publications.

Other demonstration projects have been mentioned in academic reviews with unverified and questionable efficiency claims. A recent CAES review paper cites two of these projects (Wang et al., 2017), mentioning a 1.5-MW demonstration plant with 55% efficiency and a 10-MW demonstration plant with 60% efficiency. However, verification is impossible as the web references provided are no longer available (the domains have expired) and there are no plant locations described in the article (Wang et al., 2017). Given the potential importance of these developments (a novel, large-scale thermo-mechanical energy storage system achieving in excess of 60% round-trip efficiency in a prototype system) this is very surprising. Further discussion of claims made regarding ACAES demonstration plants is available in supplemental information section S2.

## Discussion and recommendations

The idealized thermodynamic analysis undertaken illustrates some of the limitations of ACAES designs. We find that even with hypothetical, ideal components, 6.4% of the input work may be unrecoverable in a typical design. Although this does not preclude the existence of a successful plant, it highlights limits to the system performance, which have not previously been acknowledged. Furthermore, inspecting the operation required of the components, we find that an ACAES system cannot be built with off-the-shelf components. Additionally, many academic papers are restricted to theoretical and/or numerical assessments that are based on unrealistic assumptions (i.e*.,* constant compressor efficiency and HEX effectiveness), and thus over-predict achievable performance. This over-prediction is compounded by experimental studies, which report metrics such as peak-instantaneous efficiency, rather than measured round-trip efficiency. We suspect that the combination of these factors may have led to over-optimistic development in commercial ventures much too early in the research, development, and demonstration process. Ultimately, despite large amounts of funding, this has led to the failure of most ACAES commercial projects. When further considering the short timeframes for which venture capital or private investors expect a return on investment, it seems likely that ACAES is still unsuitable for commercial development without further significant breakthroughs in the technology.

Isobaric air storage is a promising option for mitigating many of the losses associated with isochoric ACAES. It removes the need for throttling, which is the major limiting factor in idealized systems, and crucially, it allows machinery to operate at design-point. This deviation from design-point operation has been identified by previous studies as a major source of inefficiency (Wang et al., 2016a; Chen et al., 2020). Several isobaric ACAES concepts have been proposed, including underwater air storage (Pimm et al., 2014; Wang et al., 2016b; Cheung et al., 2014), regulating the available storage volume by pumping fluid (Mazloum et al., 2017; Kim et al., 2011), and exploiting the phase change in a volatile fluid (Chen et al., 2018). Although any of these concepts would certainly increase the engineering complexity of the HP store, the difficulties of variable pressure operation make further research on isobaric systems worthwhile.

Future research to improve the performance of the constituent components will also be useful. For compressors, the focus should be on designs with simultaneous high single-stage compression ratios and high isentropic efficiency, with high mass flow reciprocating designs of particular interest. HEX development should focus on increasing contact area in balanced exchangers to minimize irreversibility, and further study of unsteady flow conditions in ACAES heat exchangers needs investigation (Zhang et al., 2019). Future work on systems that use packed beds for one or more of the TES may be promising, as packed beds integrate the heat exchange and thermal storage units and typically have very large contact areas for heat exchange. In particular, they may be well suited for the TES associated with the lower pressure compression stages (Zunft et al., 2017), because packed bed cost increases rapidly at higher pressures (Barbour et al., 2015). Reversible isothermal compression (Weiqing et al., 2020; Zhang et al., 2018) is another interesting avenue for future research, because near-isothermal compression and expansion would remove the need for high temperature TES. This could significantly reduce cost and allow for waste heat integration (Odukomaiya et al., 2016). However, isothermal compressors and expanders are highly experimental and have not been demonstrated at scale.

Finally, we note that history of ACAES is littered with failures and opaque claims of high performance that have subsequently turned out to be untrue. Therefore, we urge caution regarding any performance claims that are presented without clear accompanying evidence. Claims of high performance with no evidence should not be included in academic review articles without explicit mention that they are unverified. Furthermore, press releases on company websites should not be treated as verification, because these have a tendency to pre-claim results in early-stage ventures (to generate hype and investment). Most importantly, unverifiable claims should not interfere with ambitious, transparent, and much-needed experimental work on ACAES systems at universities or other publicly funded institutions. Otherwise there is a danger that *good science* on the subject will be disrupted or go unfunded, hindering progress in the long run. In general, experimental research into the performance of the constituent system components is much needed, especially under the operational conditions anticipated in real grid-scale ACAES systems. There is a particular need to improve the measured performance of heat exchangers and compressors under variable operational conditions and to explore the development of isobaric storage. Academic studies that develop prototype systems should also ensure that they publish the measured round-trip efficiency as well as modeled estimates, so the state-of-the-technology is clearly visible. Where publicly funded research is undertaken by private commercial entities or public-private research partnerships, a minimum level of documentation should be stipulated so that the lessons learned in these projects are available for future research rather than remaining hidden. In the opinion of the authors, adhering to these recommendations would significantly increase the likelihood of realizing a successful ACAES prototype.

## Methods

### Thermodynamics of compression

The work required to compress unit mass flow $\dot{m}$ of air can be estimated by considering a control volume (CV) enclosing a compressor at steady state, where the mass flow rates at the inlet and exit of the compressor are equal. The First Law of Thermodynamics yields Equation 6, where *u* is the specific internal energy ($Jkg^{-1}$), the flow work pυ is the product between pressure *p* (Pa) and specific volume υ ($m^{3}kg^{-1}$), β is the fluid velocity ($ms^{-1}$), *g* is the gravitational acceleration ($ms^{-2}$), and *z* is the height (*m*). ${\dot{Q}}^{CV}$ and ${\dot{W}}^{CV}$ are the rates of heat added to and mechanical power extracted from the control volume, respectively, given in Watts (*W*). The superscripts in and out refer to the control volume inlet and outlet respectively.

(Equation 6) $$0=\frac{{\dot{Q}}^{CV}}{\dot{m}}-\frac{{\dot{W}}^{CV}}{\dot{m}}+\left(u^{in}+p^{in}\upsilon^{in}+\frac{\beta^{in^{2}}}{2}+gz^{in}\right)-\left(u^{out}+p^{out}\upsilon^{out}+\frac{\beta^{out^{2}}}{2}+gz^{out}\right)$$

We combine the terms *u* and pυ together into specific enthalpy *h* and neglect the changes in kinetic and potential energies (typically these are much smaller in a compressor or turbine). Furthermore, we assume an adiabatic compression (${\dot{Q}}^{CV}\approx 0$) and therefore the work is given by the change in enthalpy from inlet to outlet. Approximating air as a calorically perfect gas (with constant specific heats $c_{p}$ and $c_{v}$) then the enthalpy is only a function of the temperature as shown in Equation 7.

(Equation 7) $$-\frac{{\dot{W}}^{CV}}{\dot{m}}=h^{out}-h^{in}=c_{p}\left(T^{out}-T^{in}\right)$$

For counterflow heat exchangers, the generalized energy balance neglecting thermal losses to the environment and defining an effectiveness ε (the ratio of the actual heat transfer rate to the maximum possible heat transfer ${\dot{Q}}^{MAX}$) is shown in Equation 8. The minimum heat capacity rate is defined by $C^{MIN}=\text{MIN}\left[{\dot{m}}^{c}c^{c},{\dot{m}}^{h}c^{h}\right]$, where ${\dot{m}}^{c}$ and ${\dot{m}}^{h}$ are the mass flow rates of the cold/hot fluids, respectively, and $c^{c}$ and $c^{h}$ are the cold/hot fluid heat capacities. $T^{h,in}$ and $T^{c,in}$ are the inlet temperatures of the hot and cold fluid, respectively.

(Equation 8) $$\varepsilon =\frac{\dot{Q}}{{\dot{Q}}^{MAX}}=\frac{C^{h}\left(T^{h,in}-T^{h,out}\right)}{C^{MIN}\left(T^{h,in}-T^{c,in}\right)}=\frac{C^{c}\left(T^{c,out}-T^{c,in}\right)}{C^{MIN}\left(T^{h,in}-T^{c,in}\right)}$$

In reality, the effectiveness is a function of the HEX geometry and fluid properties and can vary greatly within engineering applications (40% in automotive radiators, and in excess of 95% in gas turbine recuperators [Shah and Sekulic, 2003]), and hence will change as the pressure in the store in Figure 2 changes. However, in the limit of perfect heat exchange for balanced counterflow HEX (i.e., ε=1 and ${\dot{m}}^{c}c^{c}={\dot{m}}^{h}c^{h}$), the temperature at each compressor inlet will be equal to the ambient temperature $T^{0}$, whereas the coolant will leave the HEX at the air inlet temperature. Therefore, we can use Equation 7 to get the incremental work $\delta W^{chg}$ required to charge the HP air store at pressure *p* with a mass increment of air δm through *N* compression stages with equal compression ratio, as shown by Equation 9 and using the fact that the temperature at each compressor outlet is related to the inlet temperature by $T^{out}=T^{in}{\left(\frac{p^{out}}{p^{in}}\right)}^{\frac{\gamma -1}{N\gamma }}$, where $\gamma =\frac{c_{p}}{c_{v}}$ and the superscript ^0^ denotes the ambient state.

(Equation 9) $$\delta W^{chg}=N\delta mc_{p}T^{0}\left[{\left(\frac{p}{p^{0}}\right)}^{\frac{\gamma -1}{N\gamma }}-1\right]$$

For convenience we denote the charging work as the negative of $W^{CV}$. To relate δm to the storage pressure *p*, we differentiate the ideal gas law to obtain Equation 10:

(Equation 10) $$\frac{dp}{dm}={\left(\frac{\partial p}{\partial m}\right)}_{T}+{\left(\frac{\partial p}{\partial T}\right)}_{m}{\left(\frac{\partial T}{\partial m}\right)}_{p}.$$

The terms ${\left(\frac{\partial p}{\partial m}\right)}_{T}$ and ${\left(\frac{\partial p}{\partial T}\right)}_{m}$ are easily obtained from the ideal gas law. For the last term, ${\left(\frac{\partial T}{\partial m}\right)}_{p}$, we use the conservation of energy for the HP store, expressed by Equation 11:

(Equation 11) $$\delta mc_{p}T^{0}+mc_{v}T=\left(m+\delta m\right)c_{v}\left(T+\delta T\right)$$

Here *m* is the air mass already contained within the store, *T* is the store temperature, and δT is the change in the store temperature due to the addition of air mass δm at temperature $T^{0}$. Simplifying this leads to Equation 12.

(Equation 12) $$\frac{dT}{dm}=\frac{\gamma T^{0}-T}{m}$$

Substituting Equations 12 into 10 we find that $\frac{dp}{dm}=\frac{R\gamma T^{0}}{V^{st}}$. Further substituting this result into Equations 9 leads to 13, which gives the compression work required to raise the HP store pressure *p* from the initial charge pressure $p^{chg,i}$ to the final pressure $p^{chg,f}$.

(Equation 13) $$W^{chg}=\frac{NV^{st}c_{p}}{R\gamma }\int_{p^{chg,i}}^{p^{chg,f}}{\left(\frac{p}{p^{0}}\right)}^{\frac{\gamma -1}{N\gamma }}-1\;dp$$

Evaluating the integral leads to Equation 1. Assuming the initial storage temperature is the ambient temperature $T^{0}$, the final storage temperature is found by integrating Equation 12 and combining with the ideal gas law to give Equation 2.

### TES temperature

The TES temperature is given by the coolant temperature at the HEX outlets, which as noted above, is equal to the temperature of the air inlet in the limit of perfect HEX. Hence, the temperature of the TES is constantly changing during charge as the compressor outlet temperatures change. We assume that each TES is perfectly mixed (there is no stratification) and the heat capacity of the thermal fluid is constant. Therefore the mix temperature is the average temperature at the compressor outlet as the store is charged from $p^{chg,i}$ to $p^{chg,f}$, as shown in Equation 14 and once again using $\frac{dp}{dm}=\frac{R\gamma T^{0}}{V^{st}}$. Evaluating Equations 14 leads to 3.

(Equation 14) $$T^{TES}=\frac{\int_{0}^{m}T^{out}dm}{\int_{0}^{m}dm}=\frac{T^{0}\int_{p^{chg,i}}^{p^{chg,f}}{\left(\frac{p}{p^{0}}\right)}^{\frac{\gamma -1}{N\gamma }}dp}{\int_{p^{chg,i}}^{p^{chg,f}}dp}$$

### Thermodynamics of expansion

In Figure 2 we see that the air is throttled to $p^{thr}$ upon leaving the HP store. Therefore, the incremental discharge work ($\delta W^{dis}$) available from a mass of air δm expanded through *N* expansion stages is:

(Equation 15) $$\delta W^{dis}=\left\{ \begin{array}{ll} N\delta mc_{p}T^{TES}\left(1-{\left(\frac{p^{thr}}{p^{0}}\right)}^{\frac{1-\gamma }{N\gamma }}\right) & \text{if }p\geq p^{thr}\\ N\delta mc_{p}T^{TES}\left(1-{\left(\frac{p}{p^{0}}\right)}^{\frac{1-\gamma }{N\gamma }}\right) & \text{if }p<p^{thr} \end{array}\right.$$

Applying the conservation of energy to the store during discharge leads to $\frac{dT}{dm}=\frac{T\left(\gamma -1\right)}{m}$ and shows the store temperature can be expressed as:

(Equation 16) $$T=T^{dis,i}{\left(\frac{p}{p^{dis,i}}\right)}^{\frac{\gamma -1}{\gamma }}$$

where $T^{dis,i}$ and $p^{dis,i}$ are the initial discharge temperature and pressure respectively. Combining this with Equation 10 yields $\frac{dp}{dm}=\frac{R\gamma T}{V^{st}}$, which allows Equation 15 to be expressed with store pressure as the only variable. We further stipulate that the final discharge pressure is equal to the throttle pressure, i.e., $p^{dis,f}=p^{thr}$, which gives the available work as:

(Equation 17) $$W^{dis}=N\frac{c_{p}T^{TES}V}{R\gamma T^{dis,i}}\left[{\left(\frac{p^{thr}}{p^{0}}\right)}^{\frac{1-\gamma }{N\gamma }}-1\right]\int_{p^{thr}}^{p^{dis,i}}{\left(\frac{p^{dis,i}}{p}\right)}^{\frac{\gamma -1}{\gamma }}dp$$

Evaluating the integral in Equations 17 leads to 4.

### Limitations of the study

The results of the thermodynamic model developed are limited to a fully ideal, three-stage compression and expansion, isochoric ACAES. However, the detailed description allow for flexibility and adaptability to different conditions and layouts. The results of the present work are intended as a descriptive reference to inherent losses, as well as a generalized calculation procedure. Furthermore, no detailed design procedures for compressors, expanders, and heat exchangers are given, as this is a vast research field that could not fit under the scope of a single publication.

### Resource availability

#### Lead contact

Further information and requests for resources should be directed to the lead contact author Edward R. Barbour, E.R.Barbour@lboro.ac.uk.

#### Materials availability

This study did not generate new unique reagents or materials.

#### Data and code availability

All data needed and code developed to draw the conclusions in this paper may be requested from the authors.

## Supplemental information

Document S1. Transparent methods and Table S1
