# Supplementary material for: Why is adiabatic compressed air energy storage yet to become a viable energy storage option?
Source: iScience. 2021 Apr 16;24(5):102440. doi: 10.1016/j.isci.2021.102440 (PMC8111675; doi:10.1016/j.isci.2021.102440)
Supplement: Document S1. Transparent methods and Table S1 [file mmc1.pdf]

iScience, Volume *24*

Supplemental information

Why is adiabatic compressed air energy storage yet to become a viable energy storage option?

Edward R. Barbour, Daniel L. Pottie, and Philip Eames

S1 Exergy accounting for an ideal isochoric ACAES system - Related to section *Thermodynamic Limits*

S1.1 Charging

The idealised compression work for an N-stage (symmetrical) ACAES system (as shown in Figure 2 in the main text with $N = 3$), assuming the High Pressure (HP) air store is isochoric and adiabatic, with perfect inter-cooling Heat Exchangers (HEX) between the compression stages is given by Equation S1 (also Equation 1 in the main text).

$$W^{chg} = N\frac{V^{st}c_p p_i^{chg}}{R\gamma}\left[\frac{1}{\frac{\gamma-1}{N\gamma}+1}\left(\frac{p_f^{chg}}{p_i^{chg}}\left(\frac{p_f^{chg}}{p^{\varnothing}}\right)^{\frac{\gamma-1}{N\gamma}} - \left(\frac{p_i^{chg}}{p^{\varnothing}}\right)^{\frac{\gamma-1}{N\gamma}}\right)+1-\frac{p_f^{chg}}{p_i^{chg}}\right] \tag{S1}$$

This is the minimum input work required for ideal compressors as the store is charged and its pressure p increases from the initial pressure p_i^{chg} to the final pressure p_f^{chg}. Since the store is adiabatic, the temperature also increases from some initial temperature T_i^{chg} to a final temperature T_f^{chg}. The compression work is stored as exergy in the HP store and in the Thermal Energy Stores (TES). In the limit of lossless, balanced, counterflow HEX, the thermal energy added to the TES, by way of a Thermal Fluid (TF), has the same exergy as the air at the compressor outlets (since HEX effectiveness $\varepsilon = 1$ and $\dot{m}^{air}c^{air} = \dot{m}^{TF}c^{TF}$). We can therefore calculate the total exergy in the air added to the HP store and added to the TES.

The incremental exergy added to the HP store with a mass increment δm of air at pressure p is given by Equation S2, where h and s are the specific enthalpy and specific entropy values respectively and $h^{\varnothing}$ and $s^{\varnothing}$ are the enthalpy and entropy at the dead state respectively.

$$\delta B = \delta m\left((h-h^{\varnothing}) - T^{\varnothing}(s-s^{\varnothing})\right) \tag{S2}$$

As the air is an ideal gas and perfect HEX means that it is added to the store at the ambient temperature $T^{\varnothing}$, we can see that $h-h^{\varnothing}=0$ and the exergy added is due to the change in entropy. This entropy change for an ideal gas is $s-s^{\varnothing} = R\ln(\frac{p}{p^{\varnothing}})$. As described in the Methods section in the main text, we use $\frac{dp}{dm} = \left(\frac{\partial p}{\partial m}\right)_T + \left(\frac{\partial p}{\partial T}\right)_m \left(\frac{\partial T}{\partial m}\right)_p$ and the conservation of energy for the HP air store to find that $\frac{dp}{dm} = \frac{R\gamma T^{\varnothing}}{V^{st}}$. Using Equation S2, this allows us to express the incremental exergy added to the HP store as:

$$\delta B^{HP,add} = \delta p\frac{V^{st}}{\gamma}\ln(\frac{p}{p^{\varnothing}}) \tag{S3}$$

This is integrated to give the exergy added to the HP store as the pressure increases from the minimum pressure p_i^{chg} to the maximum pressure p_f^{chg}, as shown in Equation S4.

$$\begin{aligned} B^{HP,add} &= \frac{V^{st}}{\gamma}\int_{p_i^{chg}}^{p_f^{chg}}\ln\left(\frac{p}{p^{\varnothing}}\right)dp \\ &= \frac{V^{st}}{\gamma}\left[p_f^{chg}\ln\left(\frac{p_f^{chg}}{p^{\varnothing}}\right) - p_f^{chg} - p_i^{chg}\ln\left(\frac{p_i^{chg}}{p^{\varnothing}}\right) + p_i^{chg}\right] \end{aligned} \tag{S4}$$

The incremental exergy added to the each of the N TES is given by Equation S5, where T^{out} is the temperature at each compressor outlet and is given by $T^{in}\left(\frac{p^{out}}{p^{in}}\right)^{\frac{\gamma-1}{N\gamma}}$.

$$\delta B^{TES,add} = \delta m c_p T^{\oslash}\left(\frac{T^{out}}{T^{\oslash}} - 1 - \ln\frac{T^{out}}{T^{\oslash}}\right) \tag{S5}$$

Performing the change of variables from m to p and integrating once again yields the exergy added to the TES as shown in Equation S6. It can be easily verified that the sum of Equations S4 and S6 yields the compression work in Equation S1.

$$\begin{aligned} B^{TES,add} = \frac{NV^{st}c_p}{R\gamma}\Bigg\{ & \frac{1}{[(\gamma-1)/N\gamma]+1}\left[p_f^{chg}\left(\frac{p_f^{chg}}{p^{\oslash}}\right)^{\frac{\gamma-1}{N\gamma}} - p_i^{chg}\left(\frac{p_i^{chg}}{p^{\oslash}}\right)^{\frac{\gamma-1}{N\gamma}}\right] - \\ & - p_f^{chg}\ln\left(\frac{p_f^{chg}}{p^{\oslash}}\right)^{\frac{\gamma-1}{N\gamma}} + p_i^{chg}\ln\left(\frac{p_i^{chg}}{p^{\oslash}}\right)^{\frac{\gamma-1}{N\gamma}} - \left(\frac{\gamma-1}{N\gamma} - 1\right)\left(p_f^{chg} - p_i^{chg}\right)\Bigg\} \end{aligned} \tag{S6}$$

Since the HP air store temperature increases as the pressure rises, there will be some exergy destruction as the air entering the HP store at temperature $T^{\oslash}$ mixes with the air at variable temperature T in the store. The magnitude of this loss can be calculated by comparing the exergy change in the HP store to the exergy added (Equation S4). This exergy change is given by the final HP store exergy minus the initial store exergy as calculated using the non-flow exergy $B/m = u - u^{\oslash} + p^{\oslash}(v - v^{\oslash}) - T^{\oslash}(s - s^{\oslash})$, as expressed by Equation S7.

$$\begin{aligned} \Delta B^{HP} = \quad & p_f^{chg}V^{st}\left\{\frac{T^{\oslash}}{T_f^{chg}}\left[1 - \frac{c_v}{R} - \frac{c_p}{R}\ln\left(\frac{T_f^{chg}}{T^{\oslash}}\right) + \ln\left(\frac{p_f^{chg}}{p^{\oslash}}\right)\right] + \frac{c_v}{R} - \frac{p^{\oslash}}{p_f^{chg}}\right\} \quad - \\ & - p_i^{chg}V^{st}\left\{\frac{T^{\oslash}}{T_i^{chg}}\left[1 - \frac{c_v}{R} - \frac{c_p}{R}\ln\left(\frac{T_i^{chg}}{T^{\oslash}}\right) + \ln\left(\frac{p_i^{chg}}{p^{\oslash}}\right)\right] + \frac{c_v}{R} - \frac{p^{\oslash}}{p_i^{chg}}\right\} \end{aligned} \tag{S7}$$

The maximum temperature T_f^{chg} in the HP store can be obtained from the conservation of energy and is given by Equation S8 (Equation 2 in the main text).

$$T_f^{chg} = \frac{\gamma T^{\oslash}}{\frac{p_i^{chg}}{p_f^{chg}}(\gamma - 1) + 1} \tag{S8}$$

The exergy destroyed due to mixing in the HP air store can then be directly obtained by subtracting the Equation S7 from Equation S4.

Similarly, exergy is destroyed due to mixing in the TES since the instantaneous compressor outlet temperature depends on the variable store pressure p. To calculate the exergy destroyed due to this temperature mixing, the exergy contained within the TES at the end of the charging period can be compared to the exergy added to the TES as given by Equation S6. The temperature of the TES at the end of the charging period is given by the average temperature at the compressor outlets, shown in Equation S9 (Equation 3 in the main text).

$$T^{TES} = \frac{\int_0^m T^{out} dm}{\int_0^m dm} = \frac{T^\oslash \int_{p_i^{chg}}^{p_f^{chg}} \left(\frac{p}{p^\oslash}\right)^{\frac{\gamma-1}{N\gamma}} dp}{\int_{p_i^{chg}}^{p_f^{chg}} dp}$$

$$= \frac{T^\oslash p_i^{chg}}{\left(\frac{\gamma-1}{N\gamma}+1\right)\left(p_f^{chg}-p_i^{chg}\right)} \left[\frac{p_f^{chg}}{p_i^{chg}}\left(\frac{p_f^{chg}}{p^\oslash}\right)^{\frac{\gamma-1}{N\gamma}} - \left(\frac{p_i^{chg}}{p^\oslash}\right)^{\frac{\gamma-1}{N\gamma}}\right] \quad \text{(S9)}$$

Therefore, the exergy contained in the TES after charging is given by Equation S10, where ΔM^{chg} is the total air mass added to the HP store during the charge.

$$B^{TES} = \Delta M^{chg} c_p T^\oslash \left(\frac{T^{TES}}{T^\oslash} - 1 - \ln\frac{T^{TES}}{T^\oslash}\right) \quad \text{(S10)}$$

Thus, the exergy destroyed due to mixing in the TES is given by subtracting Equation S10 from Equation S6.

S1.2 Idle period

In the limit that there is no cooling in either the TES or the HP air store, then there will be no exergy loss during the idle period between the charge and the discharge. However, while in real systems the TES is highly insulated and will be designed to minimise thermal losses during the idle period, this is unlikely to be true for the HP air store. Therefore, we consider the effect of cooling which returns the final charging temperature of the HP air store T_f^{chg} to the ambient temperature $T^\oslash$ during the idle period. Since the store is isochoric, the pressure is directly proportional to the temperature and hence the pressure drops from p_f^{chg} to p_i^{dis}, which is the initial discharge pressure given by $p_i^{dis} = p_f^{chg}\left(\frac{T^\oslash}{T_f^{chg}}\right)$. Hence the exergy destroyed as a result of this cooling is given by:

$$\Delta B^{idle} = p_f^{chg} V^{st}\left\{\frac{T^\oslash}{T_f^{chg}}\left[1 - \frac{c_v}{R} - \frac{c_p}{R}\ln\left(\frac{T_f^{chg}}{T^\oslash}\right) + \ln\left(\frac{p_f^{chg}}{p^0}\right)\right] + \frac{c_v}{R} - \frac{p^0}{p_f^{chg}}\right\} - \\ - p_i^{dis} V^{st}\left[\frac{p^0}{p_i^{dis}} - 1 + \ln\left(\frac{p_i^{dis}}{p^0}\right)\right] \quad \text{(S11)}$$

S1.3 Discharging

The air entering the expansion train is throttled to maintain constant pressure, which allows the expanders to operate at close to their design conditions (Sciacovelli et al. 2017, Zhang et al. 2019, He et al. 2017) while the pressure in the store is greater than the throttle pressure, *i.e.* $p \geqslant p^{thr}$. If the expansion is continued with the pressure dropping below the throttle pressure then there will be a constant pressure expansion phase and a variable pressure expansion phase. In this work, we assume that in regular operation the system is designed so that the store pressure is always greater than the throttle pressure (as is the case in both existing diabatic CAES facilities in regular operation - in emergencies the pressure is allowed to reduce below the throttle pressure), hence the discharge work available from perfectly isentropic compressors is given by Equation S12 (Equation 4 in the main text).

$$W^{dis} = N\frac{c_p T^{TES} V^{st}}{RT_i^{dis}}\left[1-\left(\frac{p^{thr}}{p^{\oslash}}\right)^{\frac{1-\gamma}{N\gamma}}\right]\left[p_i^{dis} - p^{thr}\left(\frac{p^{thr}}{p_i^{dis}}\right)^{\frac{1-\gamma}{\gamma}}\right] \tag{S12}$$

Since the idealised throttling process is isenthalpic, the direct exergy destruction is a result of the change in entropy of the air as it passes through the flow restriction. This direct throttling loss can be calculated by considering the change in the flow exergy of a mass increment of air passing through the throttle valve:

$$\delta B^{thr} = \delta m R T^{\oslash} \ln\left(\frac{p}{p^{thr}}\right) \tag{S13}$$

The conservation of energy applied to the discharging process yields $\frac{dT}{dm} = \frac{T(\gamma-1)}{m}$, which in turn describes the temperature of the store during the discharge as $T = T_i^{dis}\left(\frac{p}{p_i^{dis}}\right)^{\frac{\gamma-1}{\gamma}}$. The $\frac{dT}{dm}$ term can be combined with $\frac{dp}{dm} = \left(\frac{\partial p}{\partial m}\right)_T + \left(\frac{\partial p}{\partial T}\right)_m \left(\frac{\partial T}{\partial m}\right)_p$, to yield $\frac{dp}{dm} = \frac{R\gamma T}{V^{st}}$, which allows the change of variables from store mass to store pressure in Equation S13. Thus the exergy destruction through the throttle valve during the discharge process to be calculated as:

$$\begin{aligned} \Delta B^{thr} &= \frac{V^{st} T^{\oslash}}{\gamma T_i^{dis}} \int_{p^{thr}}^{p_i^{dis}} \left(\frac{p_i^{dis}}{p}\right)^{\frac{\gamma-1}{\gamma}} \ln\left(\frac{p}{p^{thr}}\right) dp \\ &= \frac{V^{st}\gamma T^{\oslash}}{T_i^{dis}}\left[p_i^{dis}\left(\frac{1}{\gamma}\ln\left(\frac{p_i^{dis}}{p^{thr}}\right)-1\right) + p^{thr}\left(\frac{p_i^{dis}}{p^{thr}}\right)^{\frac{\gamma-1}{\gamma}}\right] \end{aligned} \tag{S14}$$

The exergy exhausted from the first heating HEX (HEX4 in Figure 2 in the main text) depends only on the inlet air temperature since we assume that all HEX are balanced and examine the limit where effectiveness $\varepsilon = 1$. Hence the incremental exergy exhausted at the first discharge HEX is given by Equation S15.

$$\delta B^{exh,HEX4} = \delta m c_p T^{\oslash}\left[\frac{T}{T^{\oslash}} - 1 - \ln\left(\frac{T}{T^{\oslash}}\right)\right] \tag{S15}$$

Using the previously developed approach and integrating between the initial and final discharge pressures yields the exergy exhausted from the HEX4 during the discharge:

$$\begin{aligned} B^{exh,HEX4} = \frac{V^{st} c_p T^{\oslash}}{R\gamma T_i^{dis}} \Bigg\{ p_i^{dis}\left(\frac{T_i^{dis}}{T^{\oslash}} - 2\gamma + \gamma^2 - \gamma \ln\frac{T_i^{dis}}{T^{\oslash}}\right) - \\ p_f^{dis}\left(\frac{T^{thr}}{T^{\oslash}} - \left(\frac{p_f^{dis}}{p_i^{dis}}\right)^{\frac{1-\gamma}{\gamma}}\left[2\gamma - \gamma^2 + \gamma \ln\left[\frac{T_i^{dis}}{T^{\oslash}}\left(\frac{p_f^{dis}}{p_i^{dis}}\right)^{\frac{\gamma-1}{\gamma}}\right]\right]\right)\Bigg\} \end{aligned} \tag{S16}$$

For the subsequent HEX (HEX5 and HEX6), the inlet temperature is equal to the previous expander outlet temperature, $T^{out} = T^{TES}\left(\frac{p^{thr}}{p^0}\right)^{\frac{1-\gamma}{N\gamma}}$. Therefore, the incremental exergy exhausted can be expressed as:

$$\delta B^{exh,HEX5} = \delta B^{exh,HEX6} = \delta m c_p T^{\varnothing} \left(\frac{T^{TES}}{T^{\varnothing}} \left(\frac{p^{thr}}{p^{\varnothing}} \right)^{\frac{1-\gamma}{N\gamma}} - 1 - \right.$$
$$\left. - \ln \left(\frac{T^{TES}}{T^{\varnothing}} \left(\frac{p^{thr}}{p^{\varnothing}} \right)^{\frac{1-\gamma}{N\gamma}} \right) \right) \tag{S17}$$

This is integrated to give Equation S18, which is also equal to the exergy in the exhaust air from the final expander.

$$B^{exh,HEX5} = \frac{V^{st} c_p T^{\varnothing}}{R T^{TES}} \left[\frac{T^{TES}}{T^{0}} \left(\frac{p^{thr}}{p^{\varnothing}} \right)^{\frac{1-\gamma}{N\gamma}} - 1 - \ln \left(\frac{T^{TES}}{T^{\varnothing}} \left(\frac{p^{thr}}{p^{\varnothing}} \right)^{\frac{1-\gamma}{N\gamma}} \right) \right]$$
$$\times \left(p_i^{dis} - p_f^{dis} \left(\frac{p_i^{dis}}{p_f^{dis}} \right)^{\frac{\gamma-1}{\gamma}} \right) \tag{S18}$$

If the mass of air extracted is less than the mass of air added to the HP store, then some Thermal Fluid (TF) will also remain in the TES units at the TES temperature (T^{TES}), since during the charge we have that $\dot{m}^{air} c_p^{air} = \dot{m}^{TF} c^{TF}$ at all times. The exergy contained in this leftover thermal fluid can be calculated from Equation S19.

$$B^{TES,remain} = N(\Delta M^{chg} - \Delta M^{dis}) c_p T^{\varnothing} \left(\frac{T^{TES}}{T^{\varnothing}} - 1 - \ln \frac{T^{TES}}{T^{\varnothing}} \right) \tag{S19}$$

Finally, the exergy remaining in the HP store is found by comparing the final non-flow exergy of the HP store with the initial exergy at the start of the charge. Equation S20 shows this:

$$B^{remain} = p_f^{dis} V^{st} \left\{ \frac{T^{\varnothing}}{T_f^{dis}} \left[1 - \frac{c_v}{R} - \frac{c_p}{R} \ln \left(\frac{T_f^{dis}}{T^{\varnothing}} \right) + \ln \left(\frac{p_i^{chg}}{p^{\varnothing}} \right) \right] + \frac{c_v}{R} - \frac{p^{\varnothing}}{p_f^{dis}} \right\}$$
$$- p_i^{chg} V^{st} \left\{ \frac{T^{\varnothing}}{T_i^{chg}} \left[1 - \frac{c_v}{R} - \frac{c_p}{R} \ln \left(\frac{T_i^{chg}}{T^{\varnothing}} \right) + \ln \left(\frac{p_i^{chg}}{p^{\varnothing}} \right) \right] + \frac{c_v}{R} - \frac{p^{\varnothing}}{p_i^{chg}} \right\} \tag{S20}$$

Thus all input and output exergy flows and exergy destruction are accounted for. Table S1 summarises this section.

S2 Current state of ACAES and performance claims - Related to section *Performance claims and current state of the technology*

The purpose of this section is to summarise our understanding of the state of development of major ACAES demonstration projects. We review several projects which include claims about the performance of experimental systems. The EU project ADELE is included since the original stated aim was the construction of an ACAES plant and it is very highly cited in the subject literature.

Exergy component	Calculation reference	Period
Compression work	Equation S1	Charging
Exergy destruction due to mixing in the HP store	Equation S4 − Equation S7	
Exergy destruction due to mixing in the TES	Equation S6 − Equation S10	
Exergy destruction due to HP store cooling	Equation S11	Idle
Direct throttling loss	Equation S14	Discharging
Exhaust exergy from the HEX4	Equation S16	
Exhaust exergy from the HEX5 & 6	$(N-1)\times$ (Equation S18)	
Turbine exhaust exergy	Equation S18	
Exergy remaining in the TES	Equation S19	
Exergy remaining in the HP store	Equation S20	

Table S1: Complete exergy accounting for the idealised ACAES systems presented in the paper.

S2.1 TICC 500 kW pilot plant

Detailed in two papers, Mei et al. (2015) and Wang, Zhang, Yang, Zhou & Wang (2016), this plant has achieved a 23% round trip efficiency (Wang, Zhang, Yang, Zhou & Wang 2016). While the first paper Mei et al. (2015) reports a higher efficiency of 33%, this appears to be a peak instantaneous efficiency inferred over a small portion of the discharge time (see Fig. 9 in Mei et al. (2015)), rather than to be reflective of measured work output over a cycle. The paper states that this efficiency was calculated *"By comparing the consumed power in the compression process and the generated power in the generation process in the same pressure variation range"*. Figure 9 in the paper clearly shows that this version of efficiency is highest when the storage pressure is lowest and the operation is closest to the design condition, highlighting the poor performance of components across a wide pressure range. This is confirmed in Wang, Zhang, Yang, Zhou & Wang (2016), wherein explaining the poor system efficiency the authors reason that *"unsteady operations could result in low efficiency of compression due to the deviation from the designed operations"*.

S2.2 Underground AA-CAES pilot-scale plant Switzerland — ALACAES

Detailed in Geissbühler et al. (2018), this study claims the "*world's first advanced adiabatic compressed air energy storage (AA-CAES) pilot-scale plant*". Actually, the study is primarily concerned with the integrity of the underground storage system which is a tunnel shaped cavern with a volume of $1,942\ m^3$. The TES is also experimentally studied. Estimated round trip efficiencies were presented since the plant neither contained a turbine, nor a suitable compression system (highlighting the lack of off-the-shelf compressors which can be used for ACAES). Instead a conventional air cooled compressor was used and thus the air was heated to $550°C$ (the estimated output temperature of an equivalent purely adiabatic compressor) with an electric heater prior to entering the cavern. The round trip efficiency was estimated in the range 63—74%, however the turbine and compressor models were basic and assumed a constant efficiency across the range of pressure ratios encountered. Hence this cannot be considered as a true demonstration plant, rather a simulation-based estimate augmented with notable experimental work on the cavern and TES. Reported investment in the facility is close to five million US dollars.

S2.3 Project ADELE

This EU funded project is consistently referenced in studies on ACAES, however despite an original mission to build the world's first large-scale ACAES demonstration plant with 70% efficiency and with reports of funding up to €10 million, no plant was ever built. Despite this, the final project documentation claims the *"main achievements include the confirmation of a round-trip efficiency of about 70%"* (Zunft et al. 2017). However, information regarding any technical details of the proposed plant design are scarce, perhaps due to legitimate concerns of commercial sensitivity. Additionally, rather than focus on the system design, a major component of the research focused on the economic case for storage in the German market (Zunft et al. 2017). While this is an important area to research, it is a far cry from building a demonstration plant of a novel thermo-mechanical energy storage system!

S2.4 SustainX

SustainX was a notable commercial isobaric ACAES venture founded in New Hampshire, USA claiming to have developed a highly efficient, near-isothermal ACAES system. Their key concept was to inject a water-air foam into the compression chamber, reducing the temperature rise during the compression and then storing the warm water. The power generation involved reversing this process by re-injecting warm foam and expanding the compressed air. The company received funding from governmental agencies and private investors, promising a rapid technology development in return. Documentation highlighted plans to build a megawatt-scale pilot plant in around three years. In 2012, a Department of Energy (US DoE) report presented the 1.5 MW pilot plant pitch, indicating that the US government invested $\$5,396,023$ in the company along with $\$7,650,565$ coming from private investors, totalling around 13 million dollars (US Department of Energy 2012). There are reports that SustainX raised more than 24 $Mi\ USD$ (Green Tech Media 2015). By August 2013, the DoE published another report, affirming that the company had completed thorough testing on a 40 kW demonstration plant and construction of the 1.5 MW prototype was underway, with the final report publication scheduled to March 2015 (US Department of energy 2013). However, when published, this report only presented preliminary results (US Department of energy 2015). On March 2015, SustainX announced that it would merge with General Compression to create GCX Energy Storage Inc. Despite promises of continuing R&D, GCX has not published any further information regarding the isothermal CAES system and it is notable that an active website is not maintained, strongly indicating that the project has ended. We could not find any scientific papers published regarding the SustainX or GCX work.

S2.5 Lightsail Energy

Founded in 2008, Lightsail Inc. was another notable early-stage commercial venture proposing to develop a near-isothermal compressed air energy storage system. Lightsail generated very significant hype with its founder, Danielle Fong, listed by Forbes magazine in 2012 as one of the world's most influential young entrepreneurs and prominent investors such as Peter Theil and Bill Gates. Lightsail's concept was the injection of water droplets into the compression chamber of a reciprocating compressor, allowing them to reach significant compression ratios with relatively little temperature increase. The warm water spray was then to be re-combined with the pressurised air and the compression processed reversed for the power generation phase. While this is a valid concept on its own, the current water-injected isothermal turbomachinery development stage is still far from commercial viability (Zhang et al. 2018). Reports suggest that Lightsail raised over 70 million dollars, including

several million dollars from the publicly-funded California Energy Commission (Green Tech Media 2016). After 10 years, the company filed for bankruptcy, and once again no significant technological development was achieved (Green Tech Media 2017). As with SustainX, Lightsail has a distinct lack of any reliable information published by dependable scientific sources, rather information is restricted to appearances in regional newspapers and internet forums.

S2.6 Hydrostor

Founded in 2010 in Toronto, Canada, Hydrostor (Hydrostor 2020*b*) is a private near-Isobaric Adiabatic Compressed Air Energy Storage company with two active projects in Canada and one under development in Australia. Their proposed system concept is based on liquid displacement in underwater ACAES systems (Wang, Xiong, Ting, Carriveau & Wang 2016) to prevent significant pressure changes during the operation cycle. As the Hydrostor ACAES charge, air is compressed and thermal energy is then removed and stored. The cool pressurised air is stored in an underwater High-Pressure air store. The inflowing air mass displaces water at near-constant hydrostatic pressure and thus the pressure variation is mitigated. This allows the compressors to run more efficiently, closer to their operational design point. When energy is required, *i.e.,* during peak-demand, air absorbs the thermal energy and is expanded in air turbines. Simultaneously, water reduces the HP store volume maintaining approximately constant pressure (Hydrostor 2020*b*). Hydrostor's first demonstration project was located in Lake Ontario, using six balloon shaped flexible structures to hold air at pressures around 0.55 *MPa* (Ebrahimi et al. 2019). This small R&D concept validation plant has operated since 2015 and is connected to the local power grid.

Hydrostor's second project is a 10 *MWh* ACAES plant in Goderich, Canada which was completed in 2019. Press releases suggest that the plant has a 2.2 *MW* compression system and 1.75 *MW* generation rated power and that the facility is connected to the Independent Electricity System Operator grid system. Its main purpose is to provide peak demand, spinning reserve, peak shaving and power and frequency regulatory services (Hydrostor 2020*b*). The company claims specific power and energy costs of $1,000 - 3,000\$/kW$ and $150 - 300\ \$/kWh$. Indications that the plant is operationally successful were published by Hydrostor later in 2019, and the project was granted the 2019 Energy Storage North America innovation award (Hydrostor 2019). Their third project is a proposed 10 *MWh* facility referred to as the "*the Angas ACAES*", located in Strathalbyn, Australia (Hydrostor 2020*a*).

While Hydrostor is success story — given its continuing operation and growing portfolio of plants — there are relatively few details available concerning the technical plant operation. Significant numerical analysis of the Toronto island plant is undertaken in Ebrahimi et al. (2019), suggesting that the storage pressure is around 550 kPa gauge. However, the actual electrical-to-electrical round-trip cycle efficiency of the plant is not stated. Using information from Table 3 in Ebrahimi et al. (2019) that relates to sensors in the real plant, it is clear that the current system efficiency is very poor. This is largely a result of the electric heater which is used to create steam to reheat the air entering the turbine (see Table 3 in Ebrahimi et al. (2019)). It is also notable that the water depth of 55 *m* restricts the operational store pressure to 550 *kPa*, which is likely to result in a relatively low stored energy capacity (the maximum volume of the balloon structures is not given). Very little information is available for the other plants. On September 2019, the company announced a further 37 *mi US*$ funding investment.

S2.7 Other ACAES demonstration projects mentioned in Review papers

There are two very notable plants which have been mentioned in the extensive review paper Wang et al. (2017). These include a ***1.5 MW demonstration project with reported*** 55% ***efficiency*** and a **10 MW demonstration project with reported efficiency in excess of** 60%. While the review paper Wang et al. (2017) implies that both of these plants are operational and the reported efficiencies are measured round-trip cycle efficiency with no supplemental heat addition, it is very difficult to verify these claims since limited other evidence exists in the public domain. The references used in the paper are to commercial websites — a company called Macaoenergy Industry — which are no longer listed as active. Other information regarding the plant is scarce and what we have found is limited to press releases from the China Energy Storage Alliance (China Energy Storage Alliance 2019). While these reiterate the efficiency claims, they are again opaque in terms of providing verification of the plant operation and performance. This is important given the extraordinary global significance of a thermo-mechanical energy storage system achieving an efficiency greater than 60% in a medium-scale pilot plant. On a further anecdotal note, the authors of this study have tried to contact several academics listed as involved with these projects, and while some email responses have been received, no further details have yet been provided.

References

China Energy Storage Alliance (2019), 'Compressed Air Energy Storage: The Path to Innovation', online. Accessed 3 Dec 2020: `http://en.cnesa.org/latest-news/2019/9/29/compressed-air-energy-storage-becoming-a-leading-energy-storage-technology`.

Ebrahimi, M., Carriveau, R., Ting, D. S.-K. & McGillis, A. (2019), 'Conventional and advanced exergy analysis of a grid connected underwater compressed air energy storage facility', *Applied Energy* **242**, 1198 – 1208.

Geissbühler, L., Becattini, V., Zanganeh, G., Zavattoni, S., Barbato, M., Haselbacher, A. & Steinfeld, A. (2018), 'Pilot-scale demonstration of advanced adiabatic compressed air energy storage, Part 1: Plant description and tests with sensible thermal-energy storage', *Journal of Energy Storage* **17**, 129–139.

Green Tech Media (2015), 'SustainX to Merge With General Compression, Abandon Above-Ground CAES Ambitions', Online. available at: https://www.greentechmedia.com/articles/read/sustainx-to-merge-with-general-compression-abandon-above-ground-caes-ambiti.

Green Tech Media (2016), 'LightSail Energy Storage and the Failure of the Founder Narrative', Online. available at: https://www.greentechmedia.com/squared/letter-from-sand-hill-road/lightsail-energy-storage-and-the-failure-of-the-founder-narrative#gs.n=k=9qI.

Green Tech Media (2017), 'LightSail Energy Enters 'Hibernation' as Quest for Game-Changing Energy Storage Runs Out of Cash', Online. available at: `https://www.greentechmedia.com/articles/read/lightsail-energy-cheap-compressed-air-storage-hibernation`.

He, W., Luo, X., Evans, D., Busby, J., Garvey, S., Parkes, D. & Wang, J. (2017), 'Exergy storage of compressed air in cavern and cavern volume estimation of the large-scale compressed air energy storage system', *Applied energy* **208**, 745–757.

Hydrostor (2019), 'Hydrostor and NRStor Announce Completion of World's First Commercial Advanced-CAES Facility', Press release. Availavle at : https://www.hydrostor.ca/news-press-1/.

Hydrostor (2020*a*), 'ANGAS A-CAES PROJECT', online. Available at : https://www.hydrostor.ca/angas-a-caes-project/.

Hydrostor (2020*b*), Hydrostor: Advanced Compressed Air Energy Storage, Technical Brochure Rev 2020, Hydrostor. available at: https://www.hydrostor.ca/wp-content/uploads/2020/01/Hydrostor_Brochure_2020.pdf.

Mei, S., Wang, J., Tian, F., Chen, L., Xue, X., Lu, Q., Zhou, Y. & Zhou, X. (2015), 'Design and engineering implementation of non-supplementary fired compressed air energy storage system: Ticc-500', *Science China Technological Sciences* **58**(4), 600–611.

Sciacovelli, A., Li, Y., Chen, H., Wu, Y., Wang, J., Garvey, S. & Ding, Y. (2017), 'Dynamic simulation of Adiabatic Compressed Air Energy Storage (A-CAES) plant with integrated thermal storage–Link between components performance and plant performance', *Applied energy* **185**, 16–28.

US Department of Energy (2012), Isothermal Compressed Air Energy Storage: Demonstrating a modular, market-ready energy storage system that uses compressed air as a storage medium, Tech report, USDoE. Available at: https://www.energy.gov/sites/prod/files/SustainX.pdf.

US Department of energy (2013), SustainX, Inc. Isothermal Compressed Air Energy Storage Fact Sheet, Tech report, USDoE. available at: https://www.energy.gov/sites/prod/files/2015/05/f22/SustainX-Isothermal-Compressed-Air-ES-Aug2013.pdf.

US Department of energy (2015), Demonstration of Isothermal Compressed Air Energy Storage to Support Renewable Energy Production, Tech report, USDoE. available at: https://www.smartgrid.gov/files/documents/Final-Technical-Report-SustainX_DE-OE0000231.pdf.

Wang, J., Lu, K., Ma, L., Wang, J., Dooner, M., Miao, S., Li, J. & Wang, D. (2017), 'Overview of compressed air energy storage and technology development', *Energies* **10**(7), 991.

Wang, S., Zhang, X., Yang, L., Zhou, Y. & Wang, J. (2016), 'Experimental study of compressed air energy storage system with thermal energy storage', *Energy* **103**, 182–191.

Wang, Z., Xiong, W., Ting, D. S.-K., Carriveau, R. & Wang, Z. (2016), 'Conventional and advanced exergy analyses of an underwater compressed air energy storage system', *Applied Energy* **180**, 810 – 822.

Zhang, W., Xue, X., Liu, F. & Mei, S. (2019), 'Modelling and experimental validation of advanced adiabatic compressed air energy storage with off-design heat exchanger', *IET Renewable Power Generation* **14**(3), 389–398.

Zhang, X., Xu, Y., Zhou, X., Zhang, Y., Li, W., Zuo, Z., Guo, H., Huang, Y. & Chen, H. (2018), 'A near-isothermal expander for isothermal compressed air energy storage system', *Applied energy* **225**, 955–964.

Zunft, S., Dreissigacker, V., Bieber, M., Banach, A., Klabunde, C. & Warweg, O. (2017), Electricity storage with adiabatic compressed air energy storage: Results of the BMWi-project ADELE-ING, *in* ‘International ETG Congress 2017’, VDE, pp. 1–5.
